# Toxicokinetic-toxicodynamic modelling of survival of *Gammarus pulex* in multiple pulse exposures to propiconazole: model assumptions, calibration data requirements and predictive power

**DOI:** 10.1007/s10646-012-0917-0

**Published:** 2012-05-05

**Authors:** Anna-Maija Nyman, Kristin Schirmer, Roman Ashauer

**Affiliations:** 1 Department of Environmental Toxicology, Eawag, Swiss Federal Institute of Aquatic Science and Technology, Überlandstrasse 133, 8600 Dübendorf, Switzerland; 2 Department of Environmental Systems Science, ETH Zürich, 8092 Zürich, Switzerland; 3 EPF Lausanne, School of Architecture, Civil and Environmental Engineering, 1015 Lausanne, Switzerland

**Keywords:** Organism recovery, Delayed toxicity, Dose response model, Pesticide risk assessment, Bioaccumulation

## Abstract

Toxicokinetic-toxicodynamic (TKTD) models quantify the time-course of internal concentration, which is defined by uptake, elimination and biotransformation (TK), and the processes which lead to the toxic effects (TD). TKTD models show potential in predicting pesticide effects in fluctuating concentrations, but the data requirements and validity of underlying model assumptions are not known. We calibrated TKTD models to predict survival of *Gammarus pulex* in propiconazole exposure and investigated the data requirements. In order to assess the need of TK in survival models, we included or excluded simulated internal concentrations based on pre-calibrated TK. Adding TK did not improve goodness of fits. Moreover, different types of calibration data could be used to model survival, which might affect model parameterization. We used two types of data for calibration: acute toxicity (standard LC50, 4 d) or pulsed toxicity data (total length 10 d). The calibration data set influenced how well the survival in the other exposure scenario was predicted (acute to pulsed scenario or vice versa). We also tested two contrasting assumptions in ecotoxicology: stochastic death and individual tolerance distribution. Neither assumption fitted to data better than the other. We observed in 10-d toxicity experiments that pulsed treatments killed more organisms than treatments with constant concentration. All treatments received the same dose, i.e. the time-weighted average concentration was equal. We studied mode of toxic action of propiconazole and it likely acts as a baseline toxicant in *G. pulex* during 10-days of exposure for the endpoint survival.

**Electronic supplementary material:**

The online version of this article (doi:10.1007/s10646-012-0917-0) contains supplementary material, which is available to authorized users.

## Introduction

Toxicokinetic-toxicodynamic (TKTD) models allow predicting pesticide effects on organisms in many exposure scenarios including fluctuating or pulsed concentrations (Ashauer et al. 2006; Ashauer et al. 2007a; Ashauer and Escher 2010; Brock et al. 2010; Jager et al. 2011; Mancini 1983; Péry et al. 2001). Toxicokinetics (TK) describe processes such as uptake, distribution, biotransformation and elimination while toxicodynamics (TD) describe the processes which lead to the effects after a compound reaches the sites of toxic action (McCarty and Mackay 1993). One of the advantages of TKTD models is the ability to predict survival of organisms upon pulsed exposure, as in pesticide applications. Aquatic environments are exposed to fluctuating pesticide concentrations, not only because pesticides are applied to fields repeatedly, but also due to natural processes which are influenced by weather (e.g. frequency and intensity of rainfall events), physico–chemical properties of the compound (e.g. hydrophobicity, stability), spills and non-agricultural point sources (Kreuger 1998; Wittmer et al. 2010).

The risks posed by a contaminant on aquatic organisms are estimated by relating the predicted environmental concentrations to effect levels (Brock et al. 2010; Traas and van Leeuwen 2007). The environmental concentrations are currently predicted using fate models (FOCUS 2001) but when comparing the exposure with the effects, different parts of the fate model output can be used, e.g. time-weighted average concentration (TWA) or initial peak concentration. However, using TWA concentrations might not always be protective for the effects of pulsed exposure. Several studies have indicated that toxic effects can be more severe in exposures to short pulses than in long constant exposure with the same TWA concentration (McCahon and Pascoe 1991; Parsons and Surgeoner 1991; Schulz and Liess 2000). The predicted environmental concentrations are compared with the effect level values such as predicted no effect concentration (PNEC) or effective concentration for 50 % of individuals in a test group (EC50) (Brock et al. 2010; Traas and van Leeuwen 2007). These methods of estimating the effects are limited because the PNEC and EC50 values become meaningless in time-varying exposure patterns (Jager 2011). For example, the time which an organism needs to eliminate the substance and recover from the damage between contaminant pulses is ignored (Ashauer and Escher 2010; Jager 2011). TKTD models can simulate survival under fluctuating exposure and they take into account internal concentrations, organism damage and recovery. Therefore, they can overcome the problems related to predicted environmental concentrations, using the fate model output as it is, and predict effect levels by simulating the effects in the corresponding exposure pattern.

Still, uncertainties related to explaining and predicting the effects of pulsed exposure remain. Two assumptions, stochastic death (SD) and individual tolerance distribution (IT), have been proposed and there are no indications that only one of them would suit all combinations of chemicals and species (Ashauer and Brown 2008; Newman and McCloskey 2000; Zhao and Newman 2007). The stochastic death hypothesis assumes death to be a random process and all individuals have equal probability to die (Bedaux and Kooijman 1994; Jager et al. 2011; Newman and McCloskey 2000; Zhao and Newman 2007). The individual tolerance hypothesis, which has been dominating the ecotoxicological theory of survival, assumes that organisms have individual effect doses (Bliss 1935; Dauterman 1994; Newman and McCloskey 2000). The two hypotheses lead to different predictions of survival in subsequent pulses of equal concentrations—the SD assumption predicts equal mortality as during the previous pulse while the IT assumption predicts no mortality during the second pulse because the individuals having low thresholds for effects were eliminated during the previous pulse. The recently developed TKTD model GUTS integrates SD and IT within one model (Jager et al. 2011). This model, including the damage, damage recovery and effect threshold, was used as a basis in the current study.

Aside from the uncertainty of the assumptions underlying SD or IT when applying TKTD models in risk assessment, there is also a lack of knowledge about the data requirements for model calibration. We conducted TK experiments, a standard 4 day LC50 test (lethal concentration for 50 % of test animals) and a 10 day pulsed toxicity experiment on *Gammarus pulex* exposed to the fungicide propiconazole. The data were used to calibrate a set of TKTD models (Jager et al. 2011). The following model assumptions and options for calibration data were investigated: (a) how does the type of calibration data influence the parameter estimation and predictive power of the survival model, (b) how well does the survival model fit to data when the TK sub-model, simulating internal concentration, is included or excluded from the survival models, and (c) does the model assuming SD or IT better describe the data?

## Materials and methods

### *Gammarus pulex* and propiconazole

*Gammarus pulex* (Crustacea, Amphipoda, Gammaridae) is a key species in aquatic environments. It feeds on leaf and other organic material and therefore plays an important role in nutrient cycling (Anderson 1979). Many fish and other aquatic species feed on *G. pulex* and therefore *G. pulex* is an important part of food webs in European streams (MacNeil et al. 1997).

Propiconazole (CAS #: 60207-90-1, log K_ow_: 3.72) is an azole fungicide which inhibits the enzyme sterol 14α-demethylase (Zarn et al. 2002). In fungi, the enzyme inhibition interferes with biosynthesis of ergosterol, which is an essential sterol component in fungal cell membranes. In animals, the enzyme is a part of the pathway leading to biosynthesis of cholesterol, which is a component of many other sterols (Zarn et al. 2002). In arthropods, one important group of cholesterol based hormones are ecdysteroids which are involved in molting: these hormones promote the replacement of the cutile (Lafont and Mathieu 2007). Therefore, even though propiconazole is a fungicide, it might act specifically in *G. pulex*. We studied the mode of toxic action of propiconazole by comparing internal concentrations of propiconazole in *G. pulex* with internal lethal concentrations (ILC_50_) of known baseline toxicants in *Daphnia magna* (Maeder et al. 2004). If the internal concentrations in *G. pulex* fall into the range of ILC_50_ values of baseline toxicants in *D. magna*, propiconazole is likely to act as a baseline toxicant (i.e. acts via narcosis) in *G. pulex* under chosen exposure conditions.

### Chemicals

A mixture of ^14^C-labelled and unlabelled propiconazole was used. The unlabelled compound (chemical purity 98.4 %) was purchased from Sigma-Aldrich and the labeled material (chemical purity 98.8 %, radiochemical purity 99.7 %) from the Institute of Isotopes Co., Ltd. Budapest, Hungary. The dosing mixture was dissolved in acetone.

### Handling of *Gammarus pulex* and exposure

The test organisms were collected from a small headwater stream in the Itziker Ried, Switzerland (E 702150, N 2360850). To acclimatize them to laboratory conditions and equalize their nutritional status, *G. pulex* were maintained for 5–7 days prior to the experiments in a large aquarium in a temperature controlled room (13 °C, 12:12 light:dark photoperiod) and fed with horse chest-nut (*Aesculus hippocastanum*) leaves which were inoculated with the fungi *Cladosporium herbarum* (Naylor et al. 1989). The water in the aquarium was pre-aerated artificial pond water (APW, Table SI-1 in the Supporting Information) (Naylor et al. 1989).

In all experiments, ten test organisms were placed 1 day prior to the start of experiments in 600 mL beakers filled with 500 mL of APW. The beakers were covered with parafilm and kept in a climate chamber (13 °C, 12:12 light:dark photoperiod). The experiments started with dosing and subsequently, the water was stirred gently with a glass rod to distribute the chemical in experimental water (carrier acetone <0.2 %). Propiconazole concentration in water was measured in every beaker directly after dosing (see below). Natural mortality and mortality caused by handling of the animals were measured using non-solvent (i.e. APW only) and solvent control beakers in addition to treated beakers. Inoculated horse-chestnut leaves were provided as leaf discs with a diameter of 20 mm and five leaf discs were given to organisms in each of the beakers. Eaten leaf discs were replaced with uncontaminated discs during the experiment. The organisms were transferred to beakers containing fresh uncontaminated APW and leaf discs, either to end an exposure period or to provide fresh APW at least once in 5 days. Water pH, conductivity and oxygen concentration were measured regularly during experiments (Tables SI-2 to SI-5 in Supporting Information).

### Design of TK experiments

The design of the TK experiments was based on Nuutinen et al. (2003) and Ashauer et al. (2010). Two TK experiments were conducted (TK1, TK2). Both included a 1-d exposure to propiconazole concentration of 7.8–9.5 nmol/mL which was below acute toxicity levels. After 1 day the animals were transferred to uncontaminated APW for 5-d (TK1) or 1-d (TK2). Eight replicate beakers were used, each containing ten *G. pulex* initially. Concentrations of propiconazole in medium were measured in every beaker (8) at different time points (TK1: 0, 5, 10, 24, 29, 34, 48, 72, 96 and 144 h and TK2: 0, 24, 29, 34 and 48 h). The average concentrations of all eight beakers per sampling time were used for modelling (Tables SI-7 and SI-9 in Supporting Information).

To determine internal concentrations of propiconazole, *G. pulex* samples were taken at the same time points as the water samples; except no *G. pulex* samples were taken at time 0 h. One *G. pulex* per beaker was taken each time, blotted dry with tissue paper and placed in a pre-weighed glass tube. Four *G. pulex* from different beakers were pooled into one sample, two pooled samples per sampling time were obtained. Pooled samples were weighed in pre-weighed glass tubes and frozen until analysis. The mean weight (±SD) of pooled samples was 84.4 ± 19.0 mg (*n* = 96 *G. pulex*, 24 pooled samples) in experiment TK1 and 108.0 ± 23.4 mg (*n* = 56 *G. pulex*, 14 pooled samples) in experiment TK2. The weight of one individual was calculated by dividing the mass of one pooled sample, containing four individuals, by four (TK 1: 21.1 ± 4.7 mg, TK 2: 27.0 ± 5.9 mg).

### Design of TD experiments

Acute toxicity of propiconazole in *G. pulex* was measured using a standard LC50 test design. The experiment consisted of seven pesticide concentrations between 8.2 and 37.4 nmol/mL (see Table SI-14 in Supporting Information) with two replicate beakers each, each beaker containing ten *G. pulex* initially. Propiconazole concentrations in water were measured and the survival of *G. pulex* was analysed by prodding and visual observation of movements daily for 4 days.

The pulsed toxicity test lasted 10 days and consisted of three treatments. Each of the treatments had seven replicate beakers, one non-solvent and one solvent control beaker. All beakers contained ten *G. pulex* initially. In two of the treatments (A, B), the organisms were exposed to two 1-d pulses (concentration around LC30, 28 nmol/mL). Between pulses, the organisms had a 2-d (A) or 6-d (B) period to recover in uncontaminated APW. In the third treatment (C), the animals were exposed constantly to the same time-weighted average (TWA) concentration as in the pulsed treatments (4.6 nmol/mL). Treatment C was conducted not only to compare the toxic effects of pulsed exposure with the corresponding constant TWA concentration but also to maximize information content for calibration of the TKTD models (Albert et al. 2012). The propicanozole concentration in water was measured and the survival was observed on a daily basis.

### Determination of aqueous chemical concentrations

In all experiments, aqueous concentrations were measured daily or more often (see sections above). A volume of 1 mL was sampled from experimental waters, 10 mL of Ecoscint A scintillation cocktail (Chemie Brunschwig, Switzerland) was added, samples were shaken and measured using a liquid scintillation counter (LSC, Tri-Carb 2200CA, Packard, USA). The counts were corrected for background activity by subtracting the activities in 10 mL Ecoscint A combined with 1 mL of uncontaminated experimental water (control beakers).

### Determination of internal concentrations

For analysis, the frozen animals were homogenized in test tubes using a glass rod. Methanol was added twice during homogenization (1 and 2.5 mL). Then, the tubes were placed into an ultrasonic bath for 5 min and the homogenate was filtered through a 0.2 μm syringe filter (regenerated cellulose). The homogenate, syringe and filter were washed two times with methanol by vortexing. The filtrate was concentrated to a suitable volume (90 μL) using GeneVac (EZ-2 PLUS, Genevac, UK) with a method of low boiling point, 60 °C, for 50 min, and under nitrogen flow. Nanopure water was added to obtain a total volume of 300 μL to establish the appropriate methanol–water ratio for high-performance liquid chromatography (HPLC) analysis of propiconazole (see Table SI-6 in Supporting Information). The samples were split and in one aliquot the concentrations were measured using a HPLC (HP 1100, Agilent) with a radiodetector (500 TR, Packard) in order to detect both parent propiconazole and its metabolites. The other aliquot was analysed with the LSC to measure the recovery of the HPLC. The recovery was on average 93 ± 18 %. In addition, the overall recovery of the sample preparation and quantification was obtained from samples of control *G. pulex* spiked with known amounts of ^14^C labelled propiconazole. The overall recovery was 72–91 %.

### Model design, formulation and description

#### TK model

Both the toxicokinetic (TK) and toxicodynamic (TD) models used here assume that the organisms do not change during the experiments. They are considered as one compartment, thus the chemical is assumed to be evenly distributed throughout the organism. A one compartment model (Eq. 1) was used to simulate TK. For this, uptake (*k*_in_) and elimination (*k*_out_) rate constants were estimated from TK data.

1 $$ \frac{{{\text{d}}C_{\text{int}} (t)}}{{{\text{d}}t}} = C_{\text{ext}} (t) \cdot k_{\text{in}} - C_{\text{int}} (t) \cdot k_{\text{out}} $$

where *C*_int_ (t) is the internal propiconazole concentration in organisms [nmol/g], *C*_ext_ (t) is the concentration in water [nmol/mL], *k*_in_ is the uptake rate constant [mL g^−1^ d^−1^], *k*_out_ is the elimination rate constant [1/d] and *t* is time [d].

#### Survival models

**Fig. 1:**
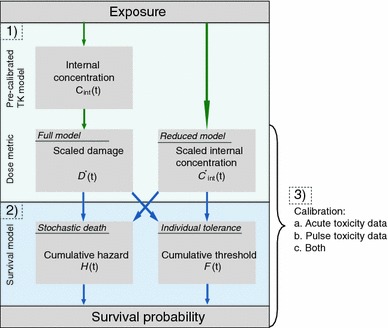
Structure of TKTD models used in this study. We tested three variations of model assumptions and data needs (numbers 1–3). First, the need of a toxicokinetic (TK) submodel was tested (*1*). Second, the assumption of survival being stochastic or deterministic for an individual was studied (*2*). Third, the data needed for calibration of the survival model was investigated (*3*)

Survival modelling was based on Jager et al. (2011). Two models assuming SD or IT were compared when pulse toxicity data, acute toxicity data or both were used to calibrate the models. SD models have one value for the threshold of survival and after exceeding it, an organism has an increased probability to die. In contrast, according to IT models the threshold is distributed within the population and death is instantaneous after exceeding the individual threshold. Both models were calibrated including and excluding the pre-calibrated TK model (full-SD, full-IT models and reduced-SD, reduced-IT models). An illustration of model types is given in Fig. 1.

#### Stochastic death models

Implementation of the stochastic death models is given in Eqs. 2–7. First (2) and (3) were used to calculate the cumulative hazard at time *t* (*H*(*t*)) when TK parameters *k*_in_ and *k*_out_ were included (full-SD). Equation 1 was used to estimate uptake and elimination rate constants prior to survival modelling by fitting Eq. 1 to the TK data alone. Then, the pre-calibrated TK model was used to simulate the internal concentrations (*C*_int_) in the survival model. Including TK in the survival model enabled the use of an explicitly modeled damage stage as dose metric for the survival probability. Thus, the TK and TD recovery processes could be distinguished, i.e. *k*_d_ in Eqn 2 describes solely the TD recovery, the elimination rate constant being estimated in Eq. 1.

2 $$ \frac{{{\text{d}}D^{*} (t)}}{{{\text{d}}t}} = k_{\text{d}} \cdot \left( {C_{\text{int}} (t) - D^{*} (t)} \right) $$

3 $$ \frac{{{\text{d}}H(t)}}{{{\text{d}}t}} = k_{\text{k}} \cdot \max (D^{*} (t) - z,0) + h_{\text{b}} (t) $$

where *D* ^*^ (t) is the scaled damage [nmol/g], *k*_d_ is the damage recovery [1/d], *k*_k_ is the killing rate [g nmol^−1^ d^−1^], *H* (t) is the cumulative hazard of an individual [−], *z* is the threshold for effects [nmol/g], *h*_b_ is the background hazard rate [1/d] (Eq. 6) and the ‘max’ function selects the maximum of either 0 or (D*(t) − *z*).

Toxicokinetics can be excluded in survival models (reduced-SD, Eqs. 4–5), but a slightly different formulation of the TD concept is needed when compared to the full-SD model (Eqs. 2–3). As the actual internal concentrations are unknown, the scaled internal concentration is used as the dose metric for the survival model (see Jager et al. 2011 for detailed explanations) and therefore the model does not include the damage stage explicitly. Instead, the dominant rate constant *k*_d_ describes both compensating processes, TK elimination and TD damage recovery. The slowest of these processes will dominate the value of *k*_d_.

4 $$ \frac{{{\text{d}}C_{\text{int}}^{*} (t)}}{{{\text{d}}t}} = k_{\text{d}} \cdot \left( {C_{\text{ext}} (t) - C_{\text{int}}^{*} (t)} \right) $$

5 $$ \frac{{{\text{d}}H(t)}}{{{\text{d}}t}} = k_{\text{k}} \cdot \max \left( {C_{\text{int}}^{*} (t) - z,0} \right) + h_{\text{b}} (t) $$

where *C*_int_^*^(t) is the scaled internal concentration [nmol/mL], *k*_d_ is the dominant rate constant [1/d], *k*_k_ is the killing rate [mL nmol^−1 ^d^−1^] and *z* is the threshold for effects [nmol/mL].

The background hazard rate *h*_b_ was obtained by fitting Eq. 6 to survival data of non-solvent and solvent controls combined.

6 $$ S_{\text{b}} = e^{{ - h_{\text{b}} t}} $$

where *S*_b_ is the background survival probability [−] describing survival in unexposed conditions.

Once the cumulative hazard *H(t)* is obtained either in the reduced or full-SD model, the survival probability, *S* (t) [−], was calculated using Eq. 7.

7 $$ S(t) = e^{ - H(t)} $$

#### Individual tolerance models

The model that assumes the threshold for death to be drawn from an individual tolerance distribution is presented in Eqs. 8–10. Reduced- and full-IT models use the same dose metrics as in SD models, scaled internal concentration *C*_int_^*^ (reduced model, Eq. 4) and scaled damage *D* ^*^ (full model, Eq. 2). Cumulative threshold distributions are based on a log-logistic cumulative distribution function (Eq. 8 for full model and Eq. 9 for reduced model). The resulting survival probability is given by Eq. 10.

8 $$ F(t) = \frac{1}{{1 + \left( {{{\mathop {\max }\limits_{0 < \tau < t} C_{\text{int}}^{*} (\tau )} \mathord{\left/ {\vphantom {{\mathop {\max }\limits_{0 < \tau < t} C_{\text{int}}^{*} (\tau )} \alpha }} \right. \kern-\nulldelimiterspace} \alpha }} \right)^{ - \beta } }} $$

9 $$ F(t) = \frac{1}{{1 + \left( {{{\mathop {\max }\limits_{0 < \tau < t} D^{*} (\tau )} \mathord{\left/ {\vphantom {{\mathop {\max }\limits_{0 < \tau < t} D^{*} (\tau )} \alpha }} \right. \kern-\nulldelimiterspace} \alpha }} \right)^{ - \beta } }} $$

10 $$ S(t) = (1 - F(t)) \cdot e^{{ - h_{\text{b}} t}} $$

where *F*(t) is the log-logistic cumulative distribution function for the threshold [−], *α* is the median of the distribution [units of dose metric, either nmol/mL for Eq. 8 or nmol/g for Eq. 9], *β* determines the width of the distribution [−] and the ‘max’ function selects the largest value of the dose metric C* or D* that occurred until time *t*.

### Model calibration

Both, the models for SD and IT were calibrated using pulse toxicity data alone, acute toxicity data alone or both data sets together. A two-step calibration was carried out. First a least squares fit using the Marquardt algorithm yielded parameter estimates. These served as initial values in the second step where the log-likelihood function (Eq. 11) (Jager et al. 2011) was maximized to find the final best fit values.

11 $$ \ln l(\theta |y) = \sum\limits_{i = 1}^{n + 1} {\left( {y_{i - 1} - y_{i} } \right)\ln \left( {S_{i - 1} (\theta ) - S_{i} (\theta )} \right)} $$

where, *l* is the likelihood for the vector of parameters *θ* given the observations *y* and *y* is the time series of the number of survivors (*y*_0_…*y*_n_).

The likelihood function compares the observed number of death events in an observation interval with the death events predicted by the model (Jager et al. 2011). Therefore, maximising the likelihood function yields the parameter set that best describes the death events over time assuming independent death events. The log-likelihoods of the treatments were added to obtain the total likelihood. The profile of log-likelihoods was used to obtain the confidence intervals (95%) for each of the parameters (Kooijman and Bedaux 1996). Modelling procedures, including run settings and initial values, are described in more detail in the SI.

In order to estimate the relative goodness of fit amongst the models and calibration data, the log-likelihood values were compared. We use the term ‘goodness of fit’ not only when the model was fitted to data by adjusting parameter values, but also when a combination of model and parameter set was used to simulate survival in another exposure scenario. Then the likelihood value was obtained by comparing the prediction with independent observations. To compare the simulation performance of each model easily between data sets and model types, we added the likelihood value of the simulation to that of the fit (shown in Fig. 4 as combined likelihood value above each model type). This is called total likelihood in the following text. In addition, the mean percentage error (MPE) was calculated (Eq. 12), because that corresponds to a practitioners view on model performance.

12 $$ {\text{MPE}} = \frac{1}{n}\sum {\frac{{\left| {S_{\text{obs}} - S_{\text{model}} } \right|}}{{S_{\text{model}} }} \cdot 100} $$

where MPE is the mean percentage error of the fraction of survivors [%], S_obs_ is the observed fraction of survivors, S_model_ is the model prediction of the fraction of survivors and n is the number of data points used in the calculation.

Survival curves in the pulsed exposure experiment were compared using the Kaplan–Meier log-rank test. The method uses the survival curves over time and compares them pairwise (e.g. Control vs Treatment C). It generates a *p* value testing the hypothesis that the survival curves are identical in the overall population.

### Model implementation

The software GraphPad Prism 4.03 (GraphPad Software Inc., San Diego, USA) was used for determination of 1-d, 2-d, 3-d and 4-d LC50 values from acute toxicity data (Least squares optimization to sigmoidal dose–response model, top fixed at 100 % and bottom fixed at 0 %) and for comparison of the survival curves in the pulsed exposure experiment (Kaplan–Meier log-rank test). For TK and TD modelling the software ModelMaker 4 (Cherwell Scientific Ltd., Oxford, UK) was used. The maximum likelihood search was implemented by minimizing—(sum of log-likelihoods, Eq. 11). The TRACE (transparent and comprehensive ecological modelling) documentation (Schmolke et al. 2010) was followed in the modelling work and is provided in the supporting information (Box SI-1).

## Results

### Toxicokinetics

**Fig. 2:**
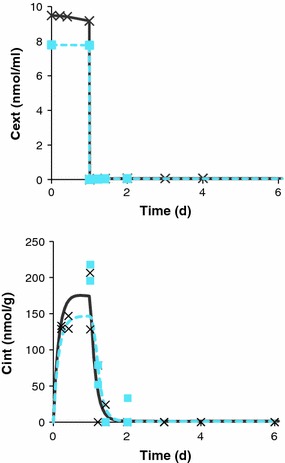
External concentration (*C*_ext_) and internal (*C*_int_) concentration of propiconazole in *Gammarus pulex*; measured in two separate toxicokinetic experiments (TK 1: *crosses*, TK 2: *squares*). The toxicokinetic model was calibrated using both data sets simultaneously (TK 1: *solid line*, TK 2: *dashed line*). Only the concentration of parent compound was used

Two possible propiconazole metabolites were observed (see Figs SI-1 and SI-2 in supporting information). Metabolite 1 appeared in 14 samples out of total 30 samples, but only in three samples was the concentration above the minimal detectable amount (MDA). The metabolite two appeared in eight samples and in none of them was the concentration above the MDA. The MDA for 1 min peaks was 78.8 dpm. As the metabolites remained mostly below levels of quantification, they were not identified. We used only the peaks of the parent compound as input for the TK model because we cannot model metabolite kinetics using only three samples. Thus *k*_out_ denotes the loss of parent propiconazole, which can occur not only via excretion or diffusion (i.e. elimination) but also via biotransformation into metabolites. Based on the TK modelling, the uptake rate constant *k*_in_ was 130.9 ± 21.9 L/(kg/d) and the elimination rate constant *k*_out_ was 6.9 ± 1.2 [1/d]. The time when 95 % of propiconazole is eliminated was calculated as 0.43 days (around 10 h). By dividing *k*_in_ by *k*_out_, the bioaccumulation factor (BAF) can be calculated, even without reaching steady state in the experiment. Based on this, propiconazole has a BAF of 19 L/kg. Dividing the internal concentration of the parent compound [nmol/g] by its external concentration [nmol/mL] after 1-d exposure yields a BAF of 22 L/kg. The time courses of external and internal concentrations are illustrated in Fig. 2 and the raw data are provided in the SI. The mortality during the TK tests was low, around 2 % in exposed and control beakers.

### Toxicodynamics

**Fig. 3:**
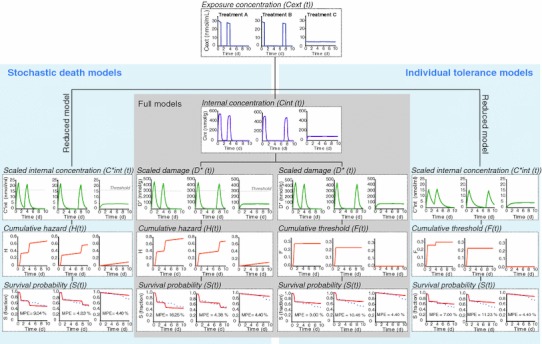
The stochastic death and individual tolerance models describing survival of *Gammarus pulex* in response to propiconazole exposure. Models are described step-by-step, starting from exposure concentrations, followed by internal concentration (full models) and dose metric illustration (scaled internal concentration/scaled damage) and survival model (cumulative hazard H/cumulative threshold F and survival fraction S). The *dots* in survival probability figures represent measured data in the pulse toxicity experiment. MPE (%) in survival graphs denotes mean percentage error. The models were calibrated using both acute and pulse toxicity data sets, however, only pulsed toxicity data is shown here (for a fit to acute toxicity data, see Fig. SI-4 in supporting information)

Based on the acute toxicity test, the LC50 values (lower–upper 95% confidence limit) were estimated as follows: 34.5 (26.5–45.0) nmol/mL after 1-d exposure, 22.5 (20.9–24.3) nmol/mL after 2-d exposure, 19.6 (18.3 to 21.0) nmol/mL after 3-d exposure and 19.2 (17.6–20.9) nmol/mL after 4-d exposure. Dose–response curves are provided in Fig SI-3 (in supporting information). In the pulse toxicity experiment, the mortality directly after the first pulse was around 20 (Tr. B) to 30 (Tr. A) %, while after the second pulse, it was only 8 (Tr A) to 9 (Tr B) % (Fig. 3). Altogether, the time-weighted average concentration did not kill as many individuals as the pulse treatments. Survival at the end of treatment A was 51 %, treatment B 53 % and in treatment C (TWA concentration) 77 % of the animals survived. Based on a survival curve analysis (Kaplan–Meier log-rank test), the differences between the treatment C and treatments A and B were significant (*p* < 0.01). In fact, the survival in treatment C did not differ significantly from that of the controls (*p* = 0.06). The survival of the controls was 90 % in the 10-d pulse toxicity experiment and 95 % in the 4-d acute toxicity test. The raw data of the pulse toxicity experiment and acute toxicity experiment, including measured exposure concentrations and number of alive organisms during the time course of the experiments, are provided in Tables SI-11 to SI-14.

**Fig. 4:**
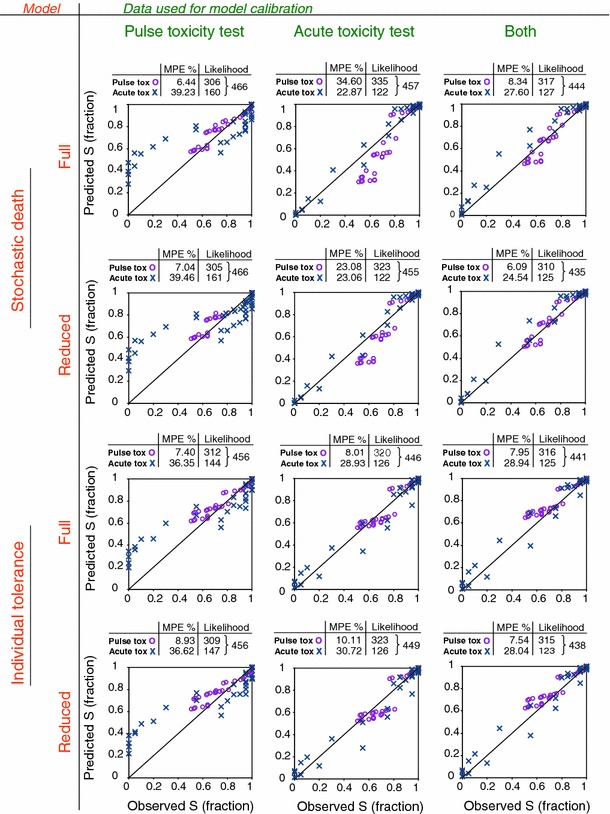
Goodness of fits among all model types and calibration data sets. ‘Full model’ denotes a model including toxicokinetics while ‘reduced model’ refers to a model excluding toxicokinetics. The models were calibrated with different data sets (acute or pulsed toxicity data or both). The observed fraction of survivors is plotted against the predicted survival. Mean predicted error (MPE, %) and likelihood values are provided above each plot. The maximum log-likelihood was implemented by minimizing—(sum of likelihoods) and therefore the smaller the value is the better is the fit

Estimates of the parameters *k*_*d*_, *k*_k_, *z*, *α* and *β* are provided in Table 1. All models (reduced-SD, full-SD, reduced-IT, full-IT) with their intermediate steps are illustrated in Fig. 3. Altogether, the SD models were difficult to calibrate using pulse toxicity data alone and therefore a modified initial parameter set and run settings were used (see Table SI-15). In addition, this combination of model and calibration data resulted in very different parameter estimates when compared with other models (Table 1). However, it cannot be concluded that the IT models better fit the data (Figs. 3, 4). A comparison of goodness of fits of the models and calibration data sets is provided in Fig. 4.

**Table 1:** Estimates of toxicodynamic parameters (lower–upper 95% confidence limit) for *Gammarus pulex* and propiconazole according to different survival models

Model		Calibration data	*k*_d_^a^	*k*_k_^b^	*z* ^c^	*α* ^d^	*β* ^e^
SD	Full	Pulsed toxicity	14.5 (4.9-n.d.)	0.0005 (0.0004–0.0007)	73.2 (31.8–81.7)	–	–
		Acute toxicity	2.7 (2.2–3.6)	0.0073 (0.0055–0.0094)	316.1 (302.2–325.2)	–	–
		Both	2.3 (2.1–2.7)	0.0051 (0.0042–0.0062)	311.6 (301.0–323.3)	–	–
	Reduced	Pulsed toxicity	5.1 (2.6–12.3)	0.0096 (0.0070–0.0125)	3.2 (1.7–4.1)	–	–
		Acute toxicity	2.1 (1.7–2.6)	0.1339 (0.1012–0.1724)	16.6 (15.8–17.1)	–	–
		Both	1.7 (1.5–1.8)	0.1260 (0.1031–0.1534)	16.4 (15.8–16.9)	–	–
IT	Full	Pulsed toxicity	0.6 (0.4–0.7)	–	–	341.1 (292.8-400.2)	2.4 (1.9–3.3)
		Acute toxicity	1.0 (0.8–1.1)	–	–	343.8 (331.5–386.2)	6.4 (5.3–9.3)
		Both	1.0 (0.9–1.1)	–	–	364.4 (350.4–378.4)	7.6 (6.1–9.3)
	Reduced	Pulsed toxicity	0.4 (0.3–0.5)	–	–	16.1 (12.2–17.8)	2.2 (1.5–2.6)
		Acute toxicity	0.9 (0.6–1.0)	–	–	18.2 (17.7–21.0)	6.5 (5.4–9.3)
		Both	0.8 (0.8–0.9)	–	–	18.7 (18.0–19.4)	7.4 (6.1–9.2)

*SD* Stochastic death model, *IT* Individual tolerance model, Full = Model including toxicokinetics, Reduced = Model excluding toxicokinetics

*n.d.* not determined (no upper limit found, must be >40)

^a^Damage recovery [1/d] (full models) or the dominant rate constant [1/d], which describes both compensating processes, TK elimination and TD damage recovery, but the slowest process dominates the value (reduced models)

^b^Killing rate [mL nmol^−1 ^d^−1^ or g nmol^−1 ^d^−1^ depending on the dose metric]

^c^Threshold for effects [nmol/mL or nmol/g depending on the dose metric]

^d^Median of threshold distribution [nmol/mL or nmol/g depending on the dose metric]

^e^Width of the distribution [−]

## Discussion

### Mode of action of propiconazole in *G. pulex*

Propiconazole inhibits the enzyme sterol 14α-demethylase which is acting in the pathway that leads to biosynthesis of cholesterol in animals and might thus interfere with molting of invertebrates (Lafont and Mathieu 2007; Zarn et al. 2002). Here, the mode of toxic action of propiconazole was studied by comparing simulated internal concentrations of propiconazole in *G. pulex* in our toxicity tests with ILC_50_ of known baseline toxicants in *Daphnia magna* (Maeder et al. 2004). The TK model was used to simulate the internal concentrations in toxicity experiments. The concentrations were corrected by lipid content, i.e. by dividing the total concentration by the amount of lipids (*G. pulex*: lipid content of 1.5 %, which was measured by a gravimetric method, unpublished data; *D. magna*: lipid content of 1.7 % (Kretschmann et al. 2011)). The simulated internal concentrations in *G. pulex* reached the lower range of the baseline toxicant ILC_50_ of *D. magna* both, in the pulsed toxicity treatments, and in the two highest concentrations of the acute toxicity test (treatments A and B) (Figs. SI-5 and SI-6 in supporting information). When the lower range of baseline ILC_50_ was reached, mortality of 20 % (pulsed toxicity test, treatment B, concentration around LC30) to 50 % (acute toxicity test, treatments A and B) was observed in our experiments. In the TWA concentration of the pulsed toxicity experiment, the internal concentrations remained far below the ILC_50_ range and accordingly, the survival curve of the treatment did not differ from that of the control. Only the second pulse in both pulsed treatments caused lower mortality than 20 %, even though simulated internal concentration reached the ILC_50_ range of the baseline toxicants. Internal lethal concentration can be calculated as BCF × LC50 (Maeder et al. 2004; McCarty and Mackay 1993), which was 43.7 μmol/g lipid for propiconazole in *G. pulex*. This falls into the *D. magna* ILC range of baseline toxicants (Maeder et al. 2004) corrected by lipid content of 1.7 % (Kretschmann et al. 2011), 35–312 μmol/g lipid. Altogether, propiconazole seems to act as a baseline toxicant in *G. pulex* in 10 d exposure for the endpoint survival. However, uncertainty remains because the baseline toxicant values that we compare with are for *D. magna* and the internal propiconazole concentrations reached only the lower ILC_50_ ranges.

### Effects of pulsed exposure

In our pulsed toxicity experiment, the treatments with two propiconazole pulses killed more individuals than the constant treatment with the corresponding TWA concentration. It has been hypothesised that the high exposure peaks cause higher bioaccumulation and thus more severe toxic effects than lower constant exposure with equivalent daily mean concentrations (Curtis et al. 1985; Parsons and Surgeoner 1991). This has been observed by Curtis and co-authors (1985) when they exposed early life-stage steelhead trout to fenvalerate (Curtis et al. 1985). Concentration dependent bioaccumulation (Liu et al. 2011) might also explain the results of this study, although we do not have direct evidence to support that hypothesis. However, permethrin caused more mortality to *A. aegypti* when the exposure was pulsed even though the exposure concentrations were equivalent. Thus, differing exposure concentrations could not explain the higher mortality by higher bioaccumulation in pulsed exposure (Parsons and Surgeoner 1991). Instead, organisms might have partially eliminated the compound and recovered between the pulses which enabled them to eat and thus take up more permethrin during the following contaminant pulse, while immobility prevented the animals from eating in constant exposure (Parsons and Surgeoner 1991; Reinert et al. 2002).

Predicted patterns of survival in multiple pulse exposures are different when based on different hypotheses of survival, stochastic death and individual tolerance (Ashauer 2010; Jager et al. 2011; Newman and McCloskey 2000; Zhao and Newman 2007). TKTD models can be used to study whether pesticide induced mortality supports IT or SD. We observed in this study with propiconazole that there appeared no clear trend between goodness of fits of SD and IT models (Fig. 4). The IT models seemed to fit better to the treatment with short recovery time between the exposure pulses while SD models described the treatment with a longer recovery period better (Fig. 3). Therefore, the hypothesis of either individual tolerance distribution or stochastic death might not solely explain the toxicity in subsequent pulses as it has been observed also by other authors (Newman and McCloskey 2000). There are other relevant processes which might affect survival (see below) and they might cause deviations from predictions provided by either of the hypothesis.

On one hand, the first pulse might weaken the surviving organisms and decrease their health, leading to increased mortality within the next pulse (Reinert et al. 2002). However, from our data we cannot infer whether the second pulse lead to “increased mortality”, because we come to opposing conclusions depending on whether we assume IT or SD (yes for IT, no for SD). In addition, our TD models would accommodate such increasing damage level. On the other hand, a first pulse might decrease mortality in the next pulse by acclimatizing the organisms to the chemical stress. For example, the first pulse might induce biotransformation and detoxification enzymes, which help the organism to deal with the subsequent pulses, and the organism might undergo some changes which alter the chemical’s target site between the pulses (Dauterman 1994; Reinert et al. 2002). Acclimatization might interfere with the two extreme hypotheses of survival and therefore neither the SD nor the IT model alone could explain the pesticide induced mortality in a pulsed exposure scenario. However, extreme cases of SD or IT could explain survival patterns in several studies. For example, a pesticide inhibiting acetylcholine esterase, diazinon, has shown clear stochastic death patterns in *G. pulex* (Ashauer et al. 2010)*.* In addition, data of mosquitofish exposed to sodium chloride or pentachlorophenol pulses supported that the stochastic component determined fish survival (Newman and McCloskey 2000). On the other hand, the support for individual tolerance theory originates in observations that insects after few generations seemed to achieve resistance to herbicides and insecticides (Bliss 1935; Dauterman 1994). Mode of toxic action as well as species characteristics might also affect the applicability of the stochastic death or individual tolerance hypothesis.

The differences between SD and IT are reflected in organism recovery times, which have been shown to be important in determining the effects of pulsed exposure (Ashauer et al. 2010; Kallander et al. 1997). Here, we calculated organism recovery times, which are defined as the time when the damage level in the organism has dropped to 5 % of the maximum after a defined pulse (Ashauer et al. 2007b; Ashauer et al. 2010). The recovery time was less than 3 days in all SD models but ranged from 4.2 to 8.5 days according to the IT models (Table 2). The differences between SD and IT are related to different model assumptions, i.e. according to IT models, organisms should not be recovered from the previous contaminant pulses in order to produce mortality during subsequent ones.

**Table 2:** Organism recovery times (95% of recovery) based on different model types

Model		Calibration data	95% recovery times (days)
SD	Full	Pulse toxicity	1.5
		Acute toxicity	2.3
		Both	2.5
	Reduced	Pulse toxicity	1.6
		Acute toxicity	2.4
		Both	2.8
IT	Full	Pulse toxicity	6.3
		Acute toxicity	4.2
		Both	4.2
	Reduced	Pulse toxicity	8.5
		Acute toxicity	4.3
		Both	4.8

*SD* Stochastic death model, *IT* Individual tolerance model, Full = Model including toxicokinetics, Reduced = Model excluding toxicokinetics

Organism recovery can be driven either by TK (i.e. elimination) or TD (i.e. damage recovery). Propiconazole was eliminated shortly after transfer to uncontaminated water (95 % elimination time ≈ 10 h). When comparing the TK and TD recovery parameters *k*_out_ and *k*_d_, the TD recovery rate constant *k*_d_ was lower than the elimination rate constant *k*_out_ according to almost all models (Table 1). Therefore, it can be concluded that TD recovery dominated overall organism recovery. Ashauer et al. (2010) came to the same conclusion when they exposed *G. pulex* to diazinon. The opposite has been observed by Ashauer et al. (2007b) when *G. pulex* were exposed to carbaryl: the elimination rate constant is 0.27 (1/d) while TD recovery rate constant is 0.97 (1/d) (Ashauer et al. 2007b), although this observation must be revised in light of new insights into biotransformation, also of carbaryl, in *G. pulex* (Ashauer et al. 2012). Ashauer et al. (2007b) measured only total radioactivity, not biotransformation. When biotransformation to naphthol-sulphate is also considered, the elimination rate of carbaryl is 2.3 (1/day) and the total loss rate for carbaryl is 5.6 (1/d) (Ashauer et al. 2012). Thus, the organism recovery of *G. pulex* exposed to carbaryl is also dominated by toxicodynamics.

### Data requirements

Previously it has been claimed that TK is an essential part of understanding survival patterns over time (Ashauer et al. 2010; Butcher et al. 2006), which might suggest that TK may also be essential for predicting survival over time. Here we compared the goodness of fit between reduced and full models (Fig. 4). The difference in model structure between the full and reduced models is that the full model includes a pre-calibrated TK sub-model which simulates internal concentrations in the different exposure scenarios (Fig. 1). This is used as an input to the survival model, where it scales the organism damage. The reduced model skips the internal concentration and damage steps but instead, the internal concentration is scaled and can be described as a lumped variable for damage and internal concentration. In almost all cases, the reduced models resulted in the same or even higher log-likelihood than the full models. This can be explained by variation which the TK experiment brings to the survival model because the TK and TD experiments were not conducted simultaneously. One can conclude that in this example, measuring and simulating TK was not essential to achieve good predictions of survival. However, even if not necessary in survival models, TK provide important information on compound bioaccumulation potential and biotransformation, as well as mode of action, and therefore should not be disregarded.

TKTD models show potential to predict effects of multiple pulse exposure. The models have been calibrated using long term pulsed toxicity experiments (Ashauer et al. 2007a, b; Ashauer et al. 2010; Butcher et al. 2006; Meyer et al. 1995) or constant exposure experiments (Mancini 1983; Meyer et al. 1995). Using the toxicity data from constant exposures for model calibration would allow applying these models widely in risk assessment because this type of data has been and is generated in standard toxicity experiments. Here, we calibrated the TKTD models using pulsed toxicity data, acute toxicity data or both together. Therefore we could compare the goodness of fit and parameter estimates amongst calibration data sets. In addition, we were able to use parameters produced by either of the data sets to predict the effects in the other scenario and compare the simulation results with observations (model validation, see TRACE in Box SI-1). The calibration data had an influence on the parameter estimates (Table 1) and on the goodness of fit (Fig. 4). As expected, overall fit was the best if both data sets were used for model calibration, i.e. the total MPE was the lowest. In addition, the total likelihood (shown in Fig. 4 as combined likelihood value for acute and pulsed toxicity data above each model type) was maximised by fitting the models to both data sets together. When only one data set was used for the calibration, the choice of calibration data affected the predictive power of the model. If we compare the total likelihood values of the models calibrated with either pulsed toxicity or acute toxicity data, we see that the maximum likelihood is achieved using acute toxicity data for calibration. Similarly, previous studies have shown that the effects of time-varying exposure can be rather well predicted based on constant acute toxicity data (Mancini 1983; Meyer et al. 1995) but the effects of constant acute toxicity exposure are poorly predicted with time-varying toxicity data (Meyer et al. 1995). Meyer et al. (1995) stated that either the models did not mimic the processes well enough or there are different physiological processes which determine the toxicity under constant and time-varying exposure. Thus, the effects of time-varying exposure might be better predicted using data from time-varying exposure under different exposure regimes than using data from constant exposure studies (Meyer et al. 1995).

The choice of calibration data also had an effect on *how* the model predicts survival in a different exposure scenario. For example, the model calibrated using pulsed toxicity data mostly underestimated mortality in the acute toxicity scenario (Fig. 4). On the other hand, when acute toxicity data alone was used to calibrate the models, mortality in the pulsed exposure scenario was overestimated. In a risk assessment context, this overestimated mortality in the prediction could be acceptable. Thus one could calibrate TKTD models using already existing acute toxicity data because they provide protective predictions of survival in a pulsed (natural) exposure scenario. However, we here studied only one combination of chemical and organism, which is not sufficient to generalize this conclusion. More evidence is required before recommendations for appropriate model calibration data or model structures (e.g. with or without TK) can be made. Therefore studies on TKTD models and how they are able to predict time-varying exposure using acute or pulsed toxicity data should be conducted using more combinations of chemicals and species.

## Electronic supplementary material

Below is the link to the electronic supplementary material.

Supplementary material 1 (DOC 995 kb)
