# Supplementary material for: Toxicokinetic-toxicodynamic modelling of survival of Gammarus pulex in multiple pulse exposures to propiconazole: model assumptions, calibration data requirements and predictive power
Source: Ecotoxicology. 2012 May 5;21(7):1828–40. doi: 10.1007/s10646-012-0917-0 (PMC3431474; doi:10.1007/s10646-012-0917-0)
Supplement: Supplementary file 1 — Supplementary material 1 (DOC 995 kb) [file 10646_2012_917_MOESM1_ESM.doc]

*Supplemental information*

Toxicokinetic-toxicodynamic modelling of survival of *Gammarus pulex* in multiple pulse exposures to propiconazole – model assumptions, calibration data requirements and predictive power

Anna-Maija Nyman ^a,b,*^, Kristin Schirmer ^a, b, c^, Roman Ashauer ^a^

^a^ Eawag, Swiss Federal Institute of Aquatic Science and Technology, Department of Environmental Toxicology, 8600 Dübendorf, Switzerland

^b^ ETH Zürich, Department of Environmental Systems Science, 8092 Zürich, Switzerland

^c^ EPF Lausanne, School of Architecture, Civil and Environmental Engineering, 1015 Lausanne, Switzerland

# * anna-maija.nyman@eawag.ch

Table of contents

[1 General 3](#__RefHeading___Toc318708793)

[1.1 Artificial pond water 3](#__RefHeading___Toc318708794)

[1.2 Water pH, conductivity and oxygen concentrations 3](#__RefHeading___Toc318708795)

[1.3 Dates when experiments were conducted 5](#__RefHeading___Toc318708796)

[2 Toxicokinetic experiments 6](#__RefHeading___Toc318708797)

[2.1 HPLC method 6](#__RefHeading___Toc318708798)

[2.2 External and internal concentrations 6](#__RefHeading___Toc318708799)

[2.3 Appearance of propiconazole metabolites in toxicokinetic experiments 8](#__RefHeading___Toc318708800)

[3 Toxicodynamic experiments 10](#__RefHeading___Toc318708801)

[3.1 Pulsed toxicity 10](#__RefHeading___Toc318708802)

[3.2 Acute toxicity 11](#__RefHeading___Toc318708803)

[3.3 Dose-response curves 12](#__RefHeading___Toc318708804)

[4 Modelling and optimization settings 13](#__RefHeading___Toc318708805)

[4.1 Least Squares and Likelihood settings in ModelMaker 4 13](#__RefHeading___Toc318708806)

[4.2 Modeling procedure 13](#__RefHeading___Toc318708807)

[4.3 An example of a survival model calibrated using all data 15](#__RefHeading___Toc318708808)

[4.4 Internal lethal concentrations (ILC_50_) of known baseline toxicants in *Daphnia magna* compared to internal concentrations of propiconazole in *Gammarus pulex*. 16](#__RefHeading___Toc318708809)

[5 TRACE documentation 18](#__RefHeading___Toc318708810)

# General

## Artificial pond water

**Table SI-1.** Composition of artificial pond water (APW) and stock solutions.

|  | Concentration in stock | Concentration in APW |
| --- | --- | --- |
| CaCl_2_. 2H_2_O (calcium chloride) | 58.80 g/L distilled water | 294.0 mg/L |
| MgSO_4_ . 7H_2_O (magnesium sulphate) | 24.65 g/L distilled water | 123.3 mg/L |
| NaHCO_3_ (sodium hydrogen carbonate) | 12.95 g/L distilled water | 64.8 mg/L |
| KCl (potassium chloride) | 1.15 g/L distilled water | 5.8 mg/L |

Artificial pond water for experiments were prepared by adding 100 ml of each stock to 20 L of nanopure water .

## Water pH, conductivity and oxygen concentrations

**Table SI-2**. The pH, conductivity and oxygen concentrations in TK 1 experiment.

| Time  [d] | Beaker | pH | Conductivity [µS/cm] | Oxygen [mg/L] |
| --- | --- | --- | --- | --- |
| 0.25 | Control | 7.47 | 574 | 6.03 |
| 0.25 | Solvent control | 7.53 | 579 | 6.21 |
| 0.25 | A1 | 7.20 | 569 | 5.54 |
| 0.25 | B3 | 7.37 | 575 | 6.18 |
| 0.25 | B4 | 7.43 | 579 | 6.97 |
| 1.25 | Control | 7.65 | 577 | 7.88 |
| 1.25 | Solvent control | 7.59 | 582 | 8.77 |
| 1.25 | A1 | 7.53 | 580 | 8.46 |
| 1.25 | A3 | 7.51 | 580 | 8.83 |
| 1.25 | B3 | 7.49 | 580 | 8.02 |

**Table SI-3**. The pH, conductivity and oxygen concentrations in TK 2 experiment.

| Time  [d] | Beaker | pH | Conductivity [µS/cm] | Oxygen [mg/L] |
| --- | --- | --- | --- | --- |
| 0.25 | Control | 6.98 | 592 | 3.64 |
| 0.25 | A1 | 7.20 | 594 | 4.95 |
| 0.25 | A3 | 7.15 | 594 | 4.20 |
| 0.25 | B1 | 7.18 | 597 | 4.83 |
| 1.25 | Control | 7.56 | 600 | 5.48 |
| 1.25 | A1 | 7.56 | 595 | 5.25 |
| 1.25 | A3 | 7.49 | 595 | 5.21 |
| 1.25 | B3 | 7.64 | 596 | 5.85 |

**Table SI-4**. The pH, conductivity and oxygen concentrations in acute toxicity experiment.

| Time  [d] | Beaker | pH | Conductivity [µS/cm] | Oxygen [mg/L] |
| --- | --- | --- | --- | --- |
| 1 | Control | 7.06 | 569 | 7.57 |
| 1 | Solvent control | 7.00 | 597 | 5.31 |
| 1 | G14 | 7.00 | 594 | 5.52 |
| 1 | D7 | 7.01 | 597 | 5.40 |
| 1 | C5 | 7.05 | 603 | 6.26 |
| 2 | Control | 6.85 | 586 | 5.21 |
| 2 | Solvent control | 6.83 | 602 | 4.29 |
| 2 | G14 | 6.81 | 595 | 4.45 |
| 2 | D8 | 6.97 | 600 | 6.29 |
| 2 | C5 | 7.00 | 605 | 6.32 |
| 3 | Control | 6.79 | 588 | 4.85 |
| 3 | Solvent control | 6.48 | 603 | 3.36 |
| 3 | G14 | 6.87 | 595 | 3.52 |
| 3 | D8 | 6.97 | 600 | 4.87 |
| 3 | C6 | 7.01 | 603 | 5.94 |
| 4 | Control | 6.84 | 599 | 3.28 |
| 4 | Solvent control | 6.86 | 610 | 3.34 |
| 4 | G13 | 6.81 | 599 | 3.46 |
| 4 | F11 | 6.77 | 597 | 2.69 |
| 4 | D7 | 6.71 | 601 | 2.60 |

**Table SI-5**. The pH, conductivity and oxygen concentrations in pulsed toxicity experiment.

| Time  [d] | Beaker | pH | Conductivity [µS/cm] | Oxygen [mg/L] |
| --- | --- | --- | --- | --- |
| 0.125 | Control A | 7.30 | 593 | 5.76 |
| 0.125 | Control B | 7.20 | 598 | 6.34 |
| 0.125 | Solvent control C | 7.18 | 588 | 6.16 |
| 0.125 | A1 | 7.11 | 597 | 5.30 |
| 0.125 | A4 | 7.18 | 599 | 6.12 |
| 0.125 | B8 | 7.15 | 601 | 5.56 |
| 0.125 | B11 | 7.22 | 598 | 6.56 |
| 0.125 | C15 | 7.28 | 593 | 5.61 |
| 0.125 | C18 | 7.21 | 586 | 5.89 |
| 6 | Control A | 7.39 | 590 | 5.25 |
| 6 | Control B | 7.35 | 590 | 5.55 |
| 6 | Control C | 7.26 | 578 | 5.45 |
| 6 | A1 | 7.37 | 586 | 5.24 |
| 6 | A4 | 7.19 | 583 | 4.65 |
| 6 | B8 | 7.36 | 591 | 5.67 |
| 6 | B11 | 7.50 | 592 | 7.12 |
| 6 | C15 | 7.24 | 589 | 5.34 |
| 6 | C18 | 7.17 | 589 | 4.88 |
| 7 | Control A | 7.31 | 594 | 4.16 |
| 7 | Control B | 7.34 | 586 | 3.66 |
| 7 | Control C | 7.28 | 587 | 3.45 |
| 7 | A1 | 7.59 | 592 | 4.39 |
| 7 | A4 | 7.34 | 590 | 3.75 |
| 7 | B8 | 7.62 | 592 | 4.87 |
| 7 | B11 | 7.58 | 592 | 4.22 |
| 7 | C15 | 7.02 | 602 | 1.90 |
| 7 | C18 | 6.90 | 595 | 2.01 |

## Dates when experiments were conducted

- Toxicokinetic 1: August 2010
- Toxicokinetic 2: September 2010
- Acute toxicity: May 2010
- Pulsed toxicity: September 2010

# Toxicokinetic experiments

## HPLC method

**Table SI-6.** HPLC method for propiconazole.

| **Column** | **Solvent A** | **Solvent B** | **Gradient** | | **Flow  (ml/min)** | **Inject. Volume** | **Wave length** | **Compound** |
| --- | --- | --- | --- | --- | --- | --- | --- | --- |
|  |  |  | **Time** | **% of B** |  |  |  |  |
| NucleodurC18 Gravity (125x2x5) | H_2_O  + 0.1% HAc | MeOH  + 0.1% HAc | 0 9 12 12.5 18 | 30 90 90 30 30 | 0.25 | 10ul | 230nm | Propiconazole (100ug/ml) |

- 1. External and internal concentrations

**Table SI-7** External concentrations of propiconazole (*C*_ext_) in the toxicokinetic experiment 1.

| **Time** | ***C*_ext_** |  |
| --- | --- | --- |
| [d] | [nmol/ml] |  |
| 0.00 | 9.48 | measured |
| 0.22 | 9.46 | measured |
| 0.41 | 9.41 | measured |
| 1.00 | 9.17 | measured |
| 1.01 | 0.00 | inserted |
| 1.21 | 0.05 | measured |
| 1.41 | 0.06 | measured |
| 2.00 | 0.08 | measured |
| 3.00 | 0.07 | measured |
| 4.00 | 0.07 | measured |
| 6.00 | 0.07 | measured |

**Table SI-8** Internal concentrations of propiconazole (*C*_int_) in the toxicokinetic experiment 1. Only the parent peaks were used in this study (highlighted with blue, parent HPLC, nmol/g). The mark ‘*below MDA’* in radio-HPLC columns denotes that we observed a peak, but it was below the minimum detectable amount, thus the concentration was taken as 0. If only 0 is given, there was no peak at all.

| **Sample** | **Sampling time** | **Total LSC** | **Parent HPLC** | **Metabolite 1 HPLC** | **Total**  **HPLC** |
| --- | --- | --- | --- | --- | --- |
|  | (d) | (nmol/g) | (nmol/g) | (nmol/g) | (nmol/g) |
| A 5h | 0.22 | 136.00 | 128.96 | 0.00 | 128.96 |
| B 5h | 0.22 | 141.55 | 133.09 | *below MDA* | 133.09 |
| A 10h | 0.41 | 176.92 | 146.74 | *below MDA* | 146.74 |
| B 10h | 0.42 | 150.10 | 129.14 | 0.00 | 129.14 |
| A 24h | 1.00 | *error ^1)^* | 206.52 | *below MDA* | 206.52 |
| B 24h | 1.00 | 180.72 | 128.32 | 24.15 | 152.47 |
| A 29h | 1.21 | 38.99 | *below MDA* | *below MDA* | 0.00 |
| B 29h | 1.21 | 105.26 | 78.48 | *below MDA* | 78.48 |
| A 34h | 1.41 | 47.72 | *below MDA* | 29.33 | 29.33 |
| B 34h | 1.42 | 52.58 | 24.12 | *below MDA* | 24.12 |
| A 48h | 2.00 | 34.58 | 0.00 | *below MDA* | 0.00 |
| B 48h | 2.00 | 25.80 | 0.00 | *below MDA* | 0.00 |
| A 72h | 3.00 | 11.56 | *below MDA* | 0.00 | 0.00 |
| B 72h | 3.01 | 6.50 | 0.00 | 0.00 | 0.00 |
| A 96h | 4.00 | 16.26 | 0.00 | *below MDA* | 0.00 |
| B 96h | 4.00 | *error ^1)^* | 0.00 | 0.00 | 0.00 |
| A 144h | 6.00 | 5.12 | 0.00 | 0.00 | 0.00 |
| B 144h | 6.00 | 5.32 | 0.00 | 0.00 | 0.00 |
| C 144h | 6.00 | 6.98 | 0.00 | *below MDA* | 0.00 |
| D 144h | 6.01 | 4.60 | 0.00 | 0.00 | 0.00 |

1) Samples were lost.

**Table SI-9** External concentrations of propiconazole (*C*_ext_) in toxicokinetic experiment 2.

| **Time** | ***C*_ext_** |  |
| --- | --- | --- |
| [d] | [nmol/ml] |  |
| 0 | 7.78 | measured |
| 1.00 | 7.77 | measured |
| 1.01 | 0.00 | inserted |
| 1.21 | 0.03 | measured |
| 1.41 | 0.05 | measured |
| 2.00 | 0.05 | measured |

**Table SI-10** Internal concentrations of propiconazole (*C*_int_) in toxicokinetic experiment 2. Only the parent peaks were used in this study (highlighted with blue, parent HPLC, nmol/g). The mark ‘*below MDA’* in radio-HPLC columns denotes that we observed a peak, but it was below the minimum detectable amount, thus the concentration was taken as 0. If only 0 is given, there was no peak at all.

| **Sample** | **Sampling time** | **Total**  **LSC** | **Parent**  **HPLC** | **Metabolite 1 HPLC** | **Total**  **HPLC** |
| --- | --- | --- | --- | --- | --- |
|  | (d) | (nmol/g) | (nmol/g) | (nmol/g) | (nmol/g) |
| A 24h | 1.00 | 263.77 | 195.41 | 32.02 | 227.43 |
| B 24h | 1.00 | 227.01 | 217.87 | *below MDA* | 217.87 |
| A 29h | 1.21 | 105.09 | 78.74 | *below MDA* | 78.74 |
| B 29h | 1.21 | 102.45 | 51.84 | *below MDA* | 51.84 |
| A 34h | 1.41 | 50.42 | *below MDA* | 0.00 | 0.00 |
| B 34h | 1.42 | 46.87 | *below MDA* | *below MDA* | 0.00 |
| A 48h | 2.00 | 26.38 | *below MDA* | 0.00 | 0.00 |
| B 48h | 2.00 | 24.90 | 0.00 | 0.00 | 0.00 |
| C 48h | 2.02 | 52.82 | 33.06 | 0.00 | 33.06 |
| D 48h | 2.02 | 16.46 | 0.00 | 0.00 | 0.00 |

## Appearance of propiconazole metabolites in toxicokinetic experiments


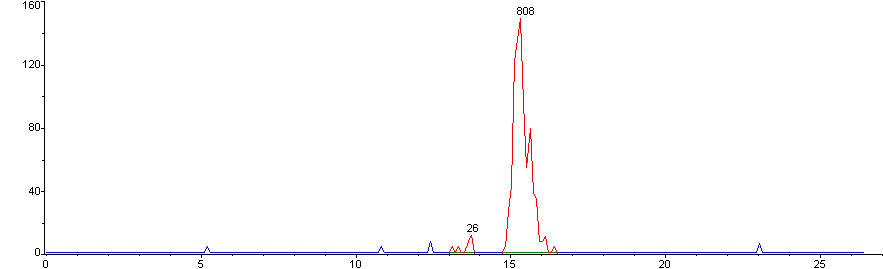


A 24-h


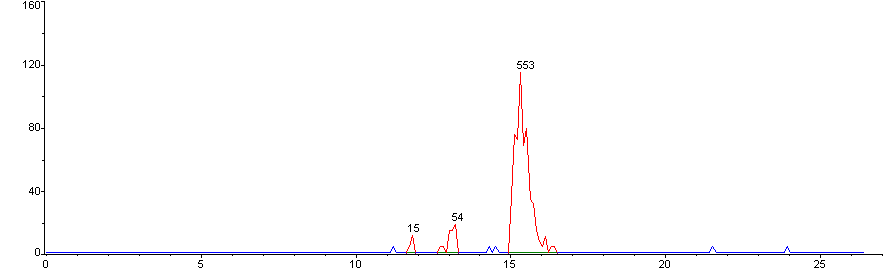


B 24-h

**Fig. SI-1.** First TK experiment: radio-HPLC chromatogram from two pooled samples after 1-d exposure. Peaks represent dpm from radio detector, the right peak is the parent propiconazole and left ones are unknown metabolites.


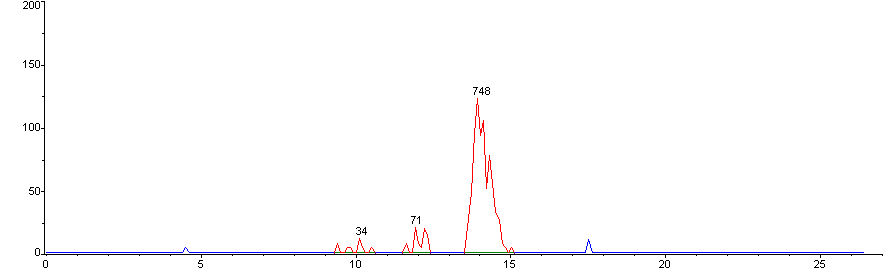


A 24-h


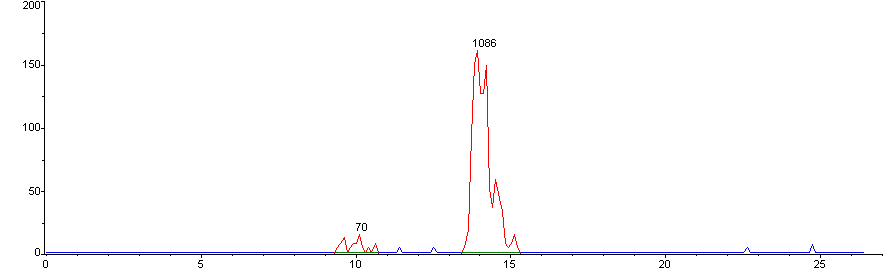


B 24-h

**Fig. SI-2.** The radio-HPLC chromatograms of the second TK experiment (two pooled samples, after 1-d exposure).

# Toxicodynamic experiments

## Pulsed toxicity

**Table SI-11.** Number of living organisms in pulsed toxicity experiment.

|  | **Treatment A** | | | | | | |  | **Treatment B** | | | | | | |  | **Treatment C** | | | | | | |  | **Solvent controls** | | |  | **Controls** | | |
| --- | --- | --- | --- | --- | --- | --- | --- | --- | --- | --- | --- | --- | --- | --- | --- | --- | --- | --- | --- | --- | --- | --- | --- | --- | --- | --- | --- | --- | --- | --- | --- |
| **Beaker** | 1 | 2 | 3 | 4 | 5 | 6 | 7 |  | 8 | 9 | 10 | 11 | 12 | 13 | 14 |  | 15 | 16 | 17 | 18 | 19 | 20 | 21 |  | 22 | 23 | 24 |  | 25 | 26 | 27 |
|  |  |  |  |  |  |  |  |  |  |  |  |  |  |  |  |  |  |  |  |  |  |  |  |  | (A) | (B) | (C) | | (A) | (B) | (C) |
| **Day** |  |  |  |  |  |  |  |  |  |  |  |  |  |  |  |  |  |  |  |  |  |  |  |  |  |  |  |  |  |  |  |
| **0** | 10 | 10 | 10 | 10 | 10 | 10 | 10 |  | 10 | 10 | 10 | 10 | 10 | 10 | 10 |  | 10 | 10 | 10 | 10 | 10 | 10 | 10 |  | 10 | 10 | 10 |  | 10 | 10 | 10 |
| **1** | 4 | 8 | 7 | 8 | 6 | 9 | 8 |  | 8 | 7 | 8 | 7 | 9 | 8 | 10 |  | 10 | 10 | 10 | 10 | 10 | 10 | 10 |  | 10 | 9 | 10 |  | 10 | 10 | 10 |
| **2** | 4 | 8 | 7 | 8 | 5 | 9 | 8 |  | 7 | 6 | 8 | 7 | 8 | 7 | 10 |  | 10 | 10 | 10 | 10 | 10 | 10 | 9 |  | 9 | 9 | 10 |  | 10 | 10 | 10 |
| **3** | 4 | 8 | 7 | 8 | 5 | 9 | 8 |  | 6 | 6 | 8 | 7 | 8 | 7 | 10 |  | 10 | 10 | 10 | 10 | 10 | 10 | 9 |  | 9 | 9 | 10 |  | 10 | 10 | 10 |
| **4** | 4 | 7 | 6 | 7 | 4 | 9 | 8 |  | 6 | 6 | 8 | 6 | 8 | 7 | 9 |  | 9 | 10 | 10 | 10 | 10 | 10 | 9 |  | 8 | 9 | 10 |  | 10 | 10 | 10 |
| **5** | 4 | 7 | 6 | 7 | 4 | 9 | 8 |  | 4 | 6 | 7 | 5 | 8 | 7 | 9 |  | 9 | 10 | 10 | 9 | 9 | 10 | 9 |  | 8 | 9 | 10 |  | 10 | 10 | 10 |
| **6** | 4 | 7 | 6 | 7 | 4 | 9 | 8 |  | 4 | 6 | 7 | 5 | 8 | 7 | 8 |  | 9 | 10 | 10 | 9 | 9 | 10 | 8 |  | 8 | 9 | 10 |  | 9 | 10 | 10 |
| **7** | 3 | 7 | 6 | 7 | 4 | 9 | 6 |  | 3 | 6 | 7 | 5 | 8 | 7 | 8 |  | 9 | 10 | 10 | 9 | 9 | 9 | 8 |  | 8 | 9 | 10 |  | 9 | 10 | 10 |
| **8** | 3 | 7 | 6 | 5 | 3 | 8 | 6 |  | 2 | 5 | 7 | 3 | 8 | 7 | 8 |  | 6 | 9 | 10 | 9 | 9 | 9 | 8 |  | 8 | 9 | 10 |  | 9 | 10 | 10 |
| **9** | 3 | 6 | 6 | 5 | 3 | 8 | 6 |  | 2 | 5 | 7 | 3 | 7 | 7 | 7 |  | 5 | 8 | 10 | 8 | 9 | 8 | 7 |  | 7 | 9 | 10 |  | 9 | 10 | 10 |
| **10** | 3 | 5 | 6 | 5 | 3 | 8 | 6 |  | 2 | 5 | 7 | 3 | 7 | 6 | 7 |  | 5 | 8 | 9 | 8 | 9 | 8 | 7 |  | 7 | 9 | 10 |  | 8 | 10 | 10 |

**Table SI-12. Exposure concentrations in pulsed toxicity experiment.**

| **Time** | **Tr. A** | **Tr. B** | **Tr. C** |
| --- | --- | --- | --- |
| [d] | [nmol/ml] | [nmol/ml] | [nmol/ml] |
| 0.00 | 30.56 | 28.98 | 4.93 |
| 0.96 | 27.93 | 27.66 | 4.69 |
| 1.00 | 0.00 | 0.00 | 4.69 |
| 1.96 | 0.26 | 0.27 | 4.58 |
| 2.96 | 0.21 | 0.26 | 4.58 |
| 3.00 | 27.69 | 0.26 | 4.58 |
| 3.96 | 26.49 | 0.26 | 4.54 |
| 4.00 | 0.00 | 0.26 | 4.54 |
| 4.96 | 0.18 | 0.25 | 4.58 |
| 4.97 | 0.18 | 0.25 | 4.71 |
| 5.96 | 0.18 | 0.03 | 4.71 |
| 6.96 | 0.14 | 0.00 | 4.60 |
| 7.00 | 0.14 | 26.98 | 4.60 |
| 7.96 | 0.18 | 26.28 | 4.59 |
| 8.00 | 0.18 | 0.00 | 4.59 |
| 9.00 | 0.00 | 0.12 | 4.46 |
| 9.96 | 0.00 | 0.12 | 4.51 |

## Acute toxicity

**Table SI-13. Survival in 4-d acute toxicity experiment.**

|  | **A** | | **B** | | **C** | | **D** | | **E** | | **F** | | **G** | | **Solvent control** | | | **Control** | | |
| --- | --- | --- | --- | --- | --- | --- | --- | --- | --- | --- | --- | --- | --- | --- | --- | --- | --- | --- | --- | --- |
| **Beaker** | 1 | 2 | 3 | 4 | 5 | 6 | 7 | 8 | 9 | 10 | 11 | 12 | 13 | 14 |  | 15 |  |  | 16 |  |
|  |  |  |  |  |  |  |  |  |  |  |  |  |  |  |  |  |  |  |  |  |
| **Day** |  |  |  |  |  |  |  |  |  |  |  |  |  |  |  |  |  |  |  |  |
| **0** | 10 | 10 | 10 | 10 | 10 | 10 | 10 | 11 | 10 | 10 | 10 | 10 | 10 | 10 |  | 10 |  |  | 10 |  |
| **1** | 1 | 10 | 4 | 7 | 8 | 9 | 10 | 11 | 10 | 9 | 9 | 10 | 10 | 10 |  | 9 |  |  | 10 |  |
| **2** | 0 | 1 | 0 | 4 | 3 | 3 | 9 | 11 | 10 | 9 | 9 | 10 | 10 | 10 |  | 9 |  |  | 10 |  |
| **3** | 0 | 0 | 0 | 0 | 2 | 0 | 6 | 10 | 10 | 8 | 9 | 10 | 10 | 10 |  | 9 |  |  | 10 |  |
| **4** | 0 | 0 | 0 | 0 | 1 | 0 | 6 | 10 | 8 | 8 | 8 | 9 | 10 | 9 |  | 9 |  |  | 10 |  |

**Table SI-14. Exposure concentrations in 4-d acute toxicity experiment.**

| **Time** | **A** | **B** | **C** | **D** | **E** | **F** | **G** |
| --- | --- | --- | --- | --- | --- | --- | --- |
| [d] | [nmol/ml] | [nmol/ml] | [nmol/ml] | [nmol/ml] | [nmol/ml] | [nmol/ml] | [nmol/ml] |
| 0 | 37.38 | 30.20 | 25.62 | 18.62 | 14.55 | 12.45 | 8.23 |
| 1 | 32.81 | 29.56 | 21.90 | 17.76 | 14.23 | 11.94 | 8.23 |
| 2 | 37.67 | 29.01 | 24.51 | 17.38 | 13.73 | 12.07 | 7.98 |
| 2.98 | 35.88 | 27.94 | 24.21 | 17.65 | 12.45 | 11.31 | 7.64 |
| 3.98 | 35.88 | 27.94 | 24.69 | 17.95 | 14.04 | 11.76 | 8.17 |

## Dose-response curves

**Fig. SI-3.** Dose-response curves in 1-4 day exposure, mortality measured in acute toxicity experiments (*Gammarus pulex*, propiconazole).

# Modelling and optimization settings

## Least Squares and Likelihood settings in ModelMaker 4

Convergence change: 0.001

Convergence steps: 50

Retry count: 10

Initial lambda: 100

Minimum change: 0.0001 (exception in full-SD model when calibrating with pulsetox data: 0.000001)

Fractional change: 0.01 (exception in full-SD model when calibrating with pulsetox data: 0.0001)

In addition in least squares fit, a grid search was conducted for initial values as multiplicative 10 with grid steps of 2.

**Least Squares method:** Optimization, Marquardt, Ordinary least squares

**Likelihood method:** Minimization of (-1 * sum_ln_likelihoods). See main manuscript (*Materials & Methods, Calibration*) and for more details of how to use the likelihood function in survival modelling.

## Modelling procedure

1. Three sets of different initial values were used in each of the models (Table SI-15). The *k*_d_ was always 0.1. The other initial parameters were adjusted to get the model lines to go through the data. Exceptions were done when SD models were calibrated using pulsed toxicity data only because the global minimum (in least squares fit) was not found with the defined initial values. Therefore, the initial values were taken from best fit of the corresponding model to LC50 data. The following steps were done for all initial parameter sets.

2. Least squares fit (optimization). Estimated parameters were written down.

3. Estimates from least squares fit were taken to likelihood model.

4. The model was run (integrated) with least squares estimate values. The sum of likelihoods was written down.

5. The least square estimates were used as initial values to maximize the sum of likelihoods by minimizing (-1 * sum_ln_likelihoods). New parameter estimates and the sum of likelihoods were written down.

6. Parameter set giving the smallest likelihood value was selected as final result (see main manuscript, *Table 1*).

**Table SI-15**. Initial values used for survival modelling. Bold values lead to the fit with the maximum likelihood.

| Model | | Calibration data | Initial set # | *k*_d_ | *k*_k_ | *z* | *α* | *β* |
| --- | --- | --- | --- | --- | --- | --- | --- | --- |
| SD- | Full | Pulse toxicity | 1 | **2.705** | **0.0073** | **316.103** | - | - |
|  |  |  | 2 | 2.727 | 0.0073 | 316.022 | - | - |
|  |  |  | 3 | 2.727 | 0.0073 | 316.467 | - | - |
|  |  | Acute toxicity | 1 | **0.1** | **0.0015** | **0** | - | - |
|  |  |  | 2 | 0.1 | 0.015 | 45 | - | - |
|  |  |  | 3 | 0.1 | 0.15 | 45 | - | - |
|  |  | Both | 1 | **0.1** | **0.0015** | **0** | - | - |
|  |  |  | 2 | 0.1 | 0.015 | 45 | - | - |
|  |  |  | 3 | 0.1 | 0.15 | 45 | - | - |
|  | Reduced | Pulse toxicity | 1 | **2.084** | **0.134** | **16.573** | - | - |
|  |  |  | 2 | 2.077 | 0.134 | 16.576 | - | - |
|  |  |  | 3 | 2.066 | 0.137 | 16.582 | - | - |
|  |  | Acute toxicity | 1 | **0.1** | **0.015** | **0** | - | - |
|  |  |  | 2 | 0.1 | 0.15 | 1.5 | - | - |
|  |  |  | 3 | 0.1 | 1.5 | 20 | - | - |
|  |  | Both | 1 | 0.1 | 0.015 | 0 | - | - |
|  |  |  | 2 | 0.1 | 0.15 | 1.5 | - | - |
|  |  |  | 3 | **0.1** | **1.5** | **20** | - | - |
| IT- | Full | Pulse toxicity | 1 | **0.1** | - | - | **75** | **4** |
|  |  |  | 2 | 0.1 | - | - | 300 | 0.75 |
|  |  |  | 3 | 0.1 | - | - | 150 | 1 |
|  |  | Acute toxicity | 1 | **0.1** | - | - | **75** | **4** |
|  |  |  | 2 | 0.1 | - | - | 300 | 0.75 |
|  |  |  | 3 | 0.1 | - | - | 150 | 1 |
|  |  | Both | 1 | **0.1** | - | - | **75** | **4** |
|  |  |  | 2 | 0.1 | - | - | 300 | 0.75 |
|  |  |  | 3 | 0.1 | - | - | 150 | 1 |
|  | Reduced | Pulse toxicity | 1 | 0.1 | - | - | 5 | 4 |
|  |  |  | 2 | **0.1** | - | - | **20** | **0.75** |
|  |  |  | 3 | 0.1 | - | - | 15 | 1 |
|  |  | Acute toxicity | 1 | 0.1 | - | - | 5 | 4 |
|  |  |  | 2 | 0.1 | - | - | 20 | 0.75 |
|  |  |  | 3 | **0.1** | - | - | **15** | **1** |
|  |  | Both | 1 | 0.1 | - | - | 5 | 4 |
|  |  |  | 2 | 0.1 | - | - | 20 | 0.75 |
|  |  |  | 3 | **0.1** | - | - | **15** | **1** |

## An example of a survival model calibrated using all data

**Fig. SI-4.** Exposure concentrations and corresponding survival fractions in acute toxicity experiment (left column, treatments A-G ) and in pulsed toxicity experiment (right column, treatments A-C). Reduced stochastic death model fit (red line for survival) is given as an example and both acute and pulsed toxicity data sets were used for calibration.

## Internal lethal concentrations (ILC_50_) of known baseline toxicants in *Daphnia magna* compared to internal concentrations of propiconazole in *Gammarus pulex*.

**Fig. SI-5**. Simulated internal concentration in *Gammarus pulex* in the pulsed toxicity experiment (blue line, left y-axis) and number of death events in pulsed toxicity experiment (red bars, right y-axis). The range of right y-axis equals to total number of organisms in the treatment (70). See exposure concentrations of the experiment in Table SI-12. Mean and lower range of internal lethal concentration (ILC_50_) for known baseline toxicants in *Daphnia magna* are based on and are given as solid and dashed green horizontal lines.

**Fig. SI-6**. Simulated internal concentration in *Gammarus pulex* in the acute toxicity experiment (blue line, left y-axis) and number of death events in acute toxicity experiment (red bars, right y-axis). The range of right y-axis equals to total number of organisms in the treatment (20). See exposure concentrations of the experiment in Table SI-14. Mean and lower range of internal lethal concentration (ILC_50_) for known baseline toxicants in *Daphnia magna* are based on and are given as solid and dashed green horizontal lines.

# TRACE documentation

**Box SI-1.** Modelling presented according to TRACE (transparent and comprehensive ecological modeling) documentation .

**1 MODEL DEVELOPMENT**

**1.1 Problem formulation**: See Introduction in the main manuscript.

**1.2 Design and formulation**: See *Materials and Methods* in main manuscript (*Model design, formulation and description*). For simplified model structure, see main manuscript Fig. 1.

**1.3 Model description**: See *Materials and Methods* in main manuscript (*Model design, formulation and description*).

**1.4 Calibration**: Experiments were carried out to obtain the data sets to calibrate the models. The experiments are described in Materials and Methods and all of the raw data are given in SI. Calibration procedure is described in main manuscript (*Model calibration*) and more details of calibration procedure including optimization settings and initial values are provided in SI. All parameters described in Model description were calibrated using corresponding data sets.

**1.5 Parameterization**: The parameters were obtained by calibration. Toxicodynamic parameters and their corresponding 95% confidence intervals are listed in main manuscript (Results, *Table 1*) and toxicokinetic parameters are provided in main manuscript, section Results*, Toxicokinetics*.

**2 MODEL TESTING AND ANALYSIS**

**2.1 Verification**: Verification was involved in structuring the models and calibration procedure. See main manuscript, *Figure 3*, in order to see whether the models work according their assumptions.

**2.2 Sensitivity analysis**: Calculating 95% confidence intervals (CIs) via likelihood profiling can be seen as a one at a time parameter sensitivity analysis. The greater the parameter range is the more insensitive the parameter is for estimating survival. Calculating the CIs is described in main manuscript (*Model calibration*) and the intervals are provided as part of parameter results (main manuscript, *Table 1*).

**2.3 Validation**: Models were validated using different data set which was used for model calibration, e.g. if model was calibrated using pulsed toxicity data, validation was done using acute toxicity data. See main manuscript, Discussion *Data requirements*, for summary of validation and Fig.4 for simulation results in comparison to validation data.

**3 MODEL APPLICATION**

**3.1 Results:** See *Table 1, Figs 2, 3* and *4* for results.

**3.2 Uncertainty analysis:** We did not carry out uncertainty analysis.

**3.3 Recommendation:** See main manuscript, *Discussion*.

References

Jager T, Albert C, Preuss TG, Ashauer R (2011) General unified threshold model of survival - a toxicokinetic-toxicodynamic framework for ecotoxicology. Environmental Science & Technology 45, 2529-2540.

Maeder V, Escher BI, Scheringer M, Hungerbühler K (2004) Toxic ratio as an indicator of the intrinsic toxicity in the assessment of persistent, bioaccumulative, and toxic chemicals. Environmental Science & Technology 38, 3659-3666.

Naylor C, Maltby L, Calow P (1989) Scope for growth in *Gammarus pulex*, a freshwater benthic detritivore. Hydrobiologia 188-189, 517-523.

Schmolke A, Thorbek P, DeAngelis DL, Grimm V (2010) Ecological models supporting environmental decision making: a strategy for the future. Trends in ecology & evolution 25, 479-486.
